# Supplementary material for: Allosteric regulation and crystallographic fragment screening of SARS-CoV-2 NSP15 endoribonuclease
Source: Nucleic Acids Res. 2023 Apr 28;51(10):5255–70. doi: 10.1093/nar/gkad314 (PMC10250223; doi:10.1093/nar/gkad314)
Supplement: gkad314_Supplemental_Files [file gkad314_supplemental_files.zip › Supplementary datasets.zip!/160/Refine_8/01-BUSTER/Cycle-5/shell.01/R_Rfree.html]

# Rxpct factors in resolution bins at cycle 0


|  |  |  |  |  |  |  |  |  |
| --- | --- | --- | --- | --- | --- | --- | --- | --- |
|  | **<d> (Å)**  Rxpct\_Work | Rxpct\_Free | Rxpct\_All | |  |  |  |  |
|  | 14.6046 | 0.3816 | 0.5491 | 0.3875 |  |
|  | 8.4320 | 0.2425 | 0.2715 | 0.2442 |  |
|  | 6.5314 | 0.2188 | 0.2626 | 0.2212 |  |
|  | 5.5200 | 0.1820 | 0.2398 | 0.1843 |  |
|  | 4.8682 | 0.1459 | 0.1988 | 0.1489 |  |
|  | 4.4035 | 0.1484 | 0.1874 | 0.1506 |  |
|  | 4.0506 | 0.1517 | 0.1868 | 0.1532 |  |
|  | 3.7709 | 0.1590 | 0.2387 | 0.1632 |  |
|  | 3.5421 | 0.1644 | 0.2016 | 0.1663 |  |
|  | 3.3505 | 0.1721 | 0.2407 | 0.1758 |  |
|  | 3.1870 | 0.1782 | 0.2345 | 0.1813 |  |
|  | 3.0453 | 0.1822 | 0.2294 | 0.1843 |  |
|  | 2.9209 | 0.1919 | 0.2184 | 0.1931 |  |
|  | 2.8107 | 0.1877 | 0.2445 | 0.1903 |  |
|  | 2.7120 | 0.1909 | 0.2168 | 0.1922 |  |
|  | 2.6231 | 0.1992 | 0.2199 | 0.2004 |  |
|  | 2.5423 | 0.2117 | 0.2193 | 0.2121 |  |
|  | 2.4686 | 0.2122 | 0.2232 | 0.2127 |  |
|  | 2.4010 | 0.2233 | 0.2313 | 0.2236 |  |
|  | 2.3386 | 0.2536 | 0.2719 | 0.2544 |  |
|  | **Overall** | 0.1879 | 0.2328 | 0.1901 |  |

# Rxpct factors in resolution bins at cycle 1


|  |  |  |  |  |  |  |  |  |
| --- | --- | --- | --- | --- | --- | --- | --- | --- |
|  | **<d> (Å)**  Rxpct\_Work | Rxpct\_Free | Rxpct\_All | |  |  |  |  |
|  | 14.6046 | 0.3816 | 0.5490 | 0.3875 |  |
|  | 8.4320 | 0.2425 | 0.2715 | 0.2442 |  |
|  | 6.5314 | 0.2188 | 0.2627 | 0.2212 |  |
|  | 5.5200 | 0.1820 | 0.2398 | 0.1844 |  |
|  | 4.8682 | 0.1459 | 0.1987 | 0.1489 |  |
|  | 4.4035 | 0.1484 | 0.1874 | 0.1506 |  |
|  | 4.0506 | 0.1517 | 0.1868 | 0.1532 |  |
|  | 3.7709 | 0.1590 | 0.2386 | 0.1632 |  |
|  | 3.5421 | 0.1644 | 0.2017 | 0.1663 |  |
|  | 3.3505 | 0.1721 | 0.2407 | 0.1758 |  |
|  | 3.1870 | 0.1782 | 0.2345 | 0.1813 |  |
|  | 3.0453 | 0.1822 | 0.2294 | 0.1843 |  |
|  | 2.9209 | 0.1919 | 0.2184 | 0.1931 |  |
|  | 2.8107 | 0.1877 | 0.2445 | 0.1903 |  |
|  | 2.7120 | 0.1908 | 0.2168 | 0.1922 |  |
|  | 2.6231 | 0.1991 | 0.2199 | 0.2004 |  |
|  | 2.5423 | 0.2117 | 0.2193 | 0.2121 |  |
|  | 2.4686 | 0.2121 | 0.2231 | 0.2127 |  |
|  | 2.4010 | 0.2232 | 0.2313 | 0.2236 |  |
|  | 2.3386 | 0.2535 | 0.2720 | 0.2544 |  |
|  | **Overall** | 0.1879 | 0.2328 | 0.1901 |  |

# Rxpct factors in resolution bins at cycle 2


|  |  |  |  |  |  |  |  |  |
| --- | --- | --- | --- | --- | --- | --- | --- | --- |
|  | **<d> (Å)**  Rxpct\_Work | Rxpct\_Free | Rxpct\_All | |  |  |  |  |
|  | 14.6046 | 0.3815 | 0.5489 | 0.3874 |  |
|  | 8.4320 | 0.2425 | 0.2714 | 0.2442 |  |
|  | 6.5314 | 0.2187 | 0.2629 | 0.2211 |  |
|  | 5.5200 | 0.1821 | 0.2397 | 0.1844 |  |
|  | 4.8682 | 0.1460 | 0.1986 | 0.1489 |  |
|  | 4.4035 | 0.1485 | 0.1874 | 0.1506 |  |
|  | 4.0506 | 0.1517 | 0.1867 | 0.1532 |  |
|  | 3.7709 | 0.1590 | 0.2385 | 0.1632 |  |
|  | 3.5421 | 0.1645 | 0.2017 | 0.1664 |  |
|  | 3.3505 | 0.1722 | 0.2407 | 0.1759 |  |
|  | 3.1870 | 0.1783 | 0.2345 | 0.1814 |  |
|  | 3.0453 | 0.1822 | 0.2294 | 0.1843 |  |
|  | 2.9209 | 0.1919 | 0.2184 | 0.1931 |  |
|  | 2.8107 | 0.1877 | 0.2445 | 0.1903 |  |
|  | 2.7120 | 0.1908 | 0.2167 | 0.1922 |  |
|  | 2.6231 | 0.1991 | 0.2198 | 0.2003 |  |
|  | 2.5423 | 0.2117 | 0.2193 | 0.2121 |  |
|  | 2.4686 | 0.2121 | 0.2230 | 0.2127 |  |
|  | 2.4010 | 0.2232 | 0.2313 | 0.2236 |  |
|  | 2.3386 | 0.2535 | 0.2718 | 0.2544 |  |
|  | **Overall** | 0.1879 | 0.2328 | 0.1901 |  |

# Rxpct factors in resolution bins at cycle 3


|  |  |  |  |  |  |  |  |  |
| --- | --- | --- | --- | --- | --- | --- | --- | --- |
|  | **<d> (Å)**  Rxpct\_Work | Rxpct\_Free | Rxpct\_All | |  |  |  |  |
|  | 14.6046 | 0.3776 | 0.5466 | 0.3835 |  |
|  | 8.4320 | 0.2400 | 0.2712 | 0.2418 |  |
|  | 6.5314 | 0.2155 | 0.2612 | 0.2180 |  |
|  | 5.5200 | 0.1812 | 0.2386 | 0.1835 |  |
|  | 4.8682 | 0.1460 | 0.2004 | 0.1490 |  |
|  | 4.4035 | 0.1488 | 0.1871 | 0.1510 |  |
|  | 4.0506 | 0.1519 | 0.1889 | 0.1535 |  |
|  | 3.7709 | 0.1589 | 0.2377 | 0.1631 |  |
|  | 3.5421 | 0.1647 | 0.2014 | 0.1665 |  |
|  | 3.3505 | 0.1723 | 0.2405 | 0.1760 |  |
|  | 3.1870 | 0.1787 | 0.2364 | 0.1819 |  |
|  | 3.0453 | 0.1823 | 0.2301 | 0.1844 |  |
|  | 2.9209 | 0.1921 | 0.2186 | 0.1933 |  |
|  | 2.8107 | 0.1878 | 0.2452 | 0.1905 |  |
|  | 2.7120 | 0.1911 | 0.2169 | 0.1925 |  |
|  | 2.6231 | 0.1990 | 0.2207 | 0.2003 |  |
|  | 2.5423 | 0.2117 | 0.2195 | 0.2121 |  |
|  | 2.4686 | 0.2120 | 0.2224 | 0.2125 |  |
|  | 2.4010 | 0.2228 | 0.2312 | 0.2232 |  |
|  | 2.3386 | 0.2532 | 0.2719 | 0.2540 |  |
|  | **Overall** | 0.1874 | 0.2329 | 0.1897 |  |
